# Novel Application of Near-infrared Spectroscopy and Chemometrics Approach for Detection of Lime Juice Adulteration

**DOI:** 10.22037/ijpr.2019.112328.13686

**Published:** 2020

**Authors:** Reza Jahani, Hassan Yazdanpanah, Saskia M. van Ruth, Farzad Kobarfard, Martin Alewijn, Arash Mahboubi, Mehrdad Faizi, Mohammad Hossein Shojaee AliAbadi, Jamshid Salamzadeh

**Affiliations:** a *Department of Toxicology and Pharmacology, School of Pharmacy, Shahid Beheshti University of Medical Sciences, Tehran, Iran. *; b *Food Safety Research Center, Shahid Beheshti University of Medical Sciences, Tehran, Iran. *; c *Wageningen Food Safety Research, Wageningen University and Research, Akkermaalsbos 2, 6708 WB, Wageningen, The Netherlands. *; d *Food Quality and Design Group, Wageningen University and Research, Bornse Weilanden 9, 6708 WG, Wageningen, The Netherlands. *; e *Department of Medicinal Chemistry, School of Pharmacy, Shahid Beheshti University of Medical Sciences, Tehran, Iran.*; f *Department of Pharmaceutics, School of Pharmacy, Shahid Beheshti University of Medical Sciences, Tehran, Iran. *; g *Faroogh Life Sciences Research Laboratory, Tehran, Iran. *; h *Department of Clinical Pharmacy, School of Pharmacy, Shahid Beheshti University of Medical Sciences, Tehran, Iran.*

**Keywords:** Lime juice, Portable NIR, Chemometrics, Food fraud, PLS-DA, k-NN

## Abstract

The aim of this study is to investigate the novel application of a ‎handheld near infra-red spectrophotometer coupled with classification methodologies as a screening approach in detection of adulterated lime juices. For this purpose, a miniaturized near infra-red spectrophotometer (Tellspec^®^) in the spectral range of 900–1700 nm was used. Three diffuse reflectance spectra of 31 pure lime juices were collected from Jahrom, Iran and 25 adulterated juices were acquired. Principal component analysis was almost able to generate two clusters. Partial least square discriminant analysis and *k*-nearest neighbors algorithms with different spectral preprocessing techniques were applied as predictive models. In the partial least squares discriminant analysis, the most accurate prediction was obtained with SNV transforming. The generated model was able to classify juices with an accuracy of 88% and the Matthew’s correlation ‎coefficient ‎value of 0.75 in the external validation set. In the *k*-NN model, the highest accuracy and Matthew’s correlation ‎coefficient in the test set (88% and 0.76, respectively) was obtained with multiplicative signal correction followed by 2^nd^-order derivative and 5^th^ nearest neighbor. The results of this preliminary study provided promising evidence of the potential of the handheld near infra-red spectrometer and machine learning methods for rapid detection of lime juice adulteration. Since a limited number of the samples were used in the current study, more lime juice samples from a wider range of variability need to be analyzed in order to increase the robustness of the generated models and to confirm the promising results achieved in this study.

## Introduction

Lime is commercialized in the market as fresh fruit, juice, and oil (1). Titratable acidity and citric acid concentration are two main factors which affect the price of lime juice and its concentrate. Thus, adulteration is simply performed by the addition of water, sugar, citric acid and/or other acidifying agents (2). Several methods and techniques were used to evaluate and guarantee lime and lemon juice authenticity. High-performance liquid chromatography, enzymatic method and capillary isotachophoresis, mass spectroscopy, and isotope ratio mass spectroscopy are some examples (1, 3-7). Different methods were often used to measure the concentration of citric acid, isocitric acid, and their ratio as one of the main parameters in the detection of lime juice adulteration (5). An LC-MS/MS method has also been developed by the FDA to identify adulteration of lemon juice by water dilution (7). The capability of isotope ratio mass spectroscopy in the detection of lemon juice adulteration was evaluated by Guyon *et al. *(1). These methods have some shortcomings such as high expenses; therefore, they cannot be used in all laboratories. Moreover, they are cumbersome, laboratory-based, and not quick enough for analyzing a large number of samples in a short period of time. Therefore, they cannot be applied as screening methods. 

Among different techniques, near infra-red spectroscopy (NIRS) was used as a rapid, low-cost, convenient, precise, multi-analytical, and non-destructive screening method for food authentication (8, 9). Recently, the ability of combined data mining/NIRS for purity assessment of lime juice using a benchtop NIRS was reported by Shafiee *et al.* (10). Detection of olive oil adulterated with other vegetable oils, melamine in milk, milk powder, and soya bean meal and the adulteration of spices with low-cost ingredients are some other applications of the NIRS in the food adulteration area (11, 12). Moreover, several research studies in the last years have revealed the application of portable NIRS in food sciences as a tool for rapid analysis of various food matrices. Some examples are the application of portable NIRS for organic milk authentication, rapid analysis of rice authenticity, and salted minced meat composition diagnostics (13-15).

NIRS can be applied in order to acquire qualitative and/or quantitative information coming from multiple organic components based on the electromagnetic absorption in the short wavelength infrared range (780–2500 nm) (16). The physicochemical detailed information contained within the wavelength spectrum, that is either absorbed or emitted, can be provided by the NIRS through interaction between electromagnetic radiation (in the energy range of 2.65 × 10^-19^ to 7.96 × 10^-20^ J) and the sample atoms and molecules (17). NIRS is able to detect all organic compounds rich in O–H bonds, C–H bonds, and N–H bonds. This makes it possible to identify functional groups in a sample. The complex relationship between the intensity of absorption and wavelength in the spectral range due to overtones and combination bands of O–H, N–H, C–H, and S–H stretching and bending vibrations is exclusive to each matrix. This complex relationship is considered as the fingerprint of that matrix (18). 

Sometimes these fingerprints contain over 1,000 spectral variables related to the physicochemical composition of the sample in their own unique way. Chemometrics helps scientists obtain reliable results in different ambits of food science and food-related issues. Multivariate classification techniques could be performed to extract the relevant part of multivariate NIR spectral data without losing important information which can affect final predictions or measurements and to get rid of useless variables (*e.g.*, interferences or noise). Indeed, modeling techniques such as principal component analysis (PCA), partial least squares-discriminant analysis (PLS-DA), and *k*-nearest neighbors (*k*-NN) can provide an interpretable and reliable connection among variables describing food composition (19). PLS-DA is a simple, robust, linear, and interpretable algorithm. In addition, various statistic parameters such as loading weight, variable importance on projection (VIP), and regression coefficient are provided by this algorithm that could be applied in the identification of the most important variables (20). *K*-NN algorithm is also very simple to understand and equally easy to implement. There are only a few parameters such as distance metric and k value that need to be tuned. *K*-NN does not explicitly build any model. It simply tags the new data entry based learning from historical data. This is a good classification model even if the classes are not linearly separable (21). However, it is very important to find the classification algorithms and preprocessing techniques that have the highest reliable sensitivity, specificity, and accuracy (22). Therefore, the aim of this study is to investigate the novel application of a handheld‎ NIRS in combination with classification methodologies as a screening method for the rapid detection of lime juice adulteration.

## Experimental

*Sample collection and preparation*

A total of 31 samples of lime fruit‎ (*Citrus latifolia*‏‎) originated from Jahrom city, IR. Iran were directly obtained from the local market of Tehran, IR Iran between April and December 2018. Lime fruit samples were gently squeezed by a manual citrus juicer (MCP 3500, Bosch, Germany), and homogenized using the Ultra-Turrax homogenizer (T8; IKA, Staufen, Germany). Twenty-five adulterated lime juice samples were kindly donated by the Iranian Food and Drug Administration. These samples were detected as adulterated samples based on citric acid to iso-citric acid ratio. The samples with a citric acid to iso-citric acid ratio over 300 were considered as non-genuine samples (23). In these samples, adulteration was performed by the addition of water and subsequently citric acid as an acidifying agent.

*Spectral collection (Portable NIRS)*

A miniaturized research model NIRS device (Tellspec^®^, Tellspec Inc., Toronto, Canada) connected to a smartphone was used in this study. Tellspec is equipped with two integrated halogen tungsten lamps and a single 1mm InGaAs detector on the same side which makes it able to operate as a diffuse reflectance NIRS. Exposure time, wavelength resolution, and accuracy were 0.635 ms, 12 nm and 2 nm, respectively (15, 24). Three diffuse reflectance spectra at three random spots were acquired for each sample in the spectral range of 900–1700 nm (11,111-5,882 cm^-1^) which included 256 points with 3 nm spectral steps. The averaged spectra of three acquired scans from each sample were subsequently used for fingerprinting and data elaboration.

*Statistical analysis*

*Data preprocessing*

Different preprocessing techniques including multiplicative scatter correction (MSC), standard normal variate (SNV), and 2^nd^-order derivative (2^nd^-Dv) were conducted on the whole spectra of genuine and adulterated juices before performing unsupervised and supervised algorithms. These preprocessing techniques were applied as they are the most widely used algorithms in NIRS in both reflectance and transmittance mode.

*Principal component analysis*

In order to visualize a description of the dataset, a multivariate statistical analysis was performed on the dataset, and different preprocessing techniques were conducted on the whole spectra of genuine and adulterated juices to find out which preprocessing technique could discriminate adulterated samples from genuine ones. PCA as a dimension-reduction tool was used to reduce the number of variables. PCA transforms the correlated variables into the uncorrelated variables called principal components (25). To find out the variables which were more important in sample clustering, PC score plot was generated.

*Partial Least Squares Discriminant Analysis*

For sample clustering and making predictive models based on the state of adulteration, PLS-DA classifier was used to distinguish the different groups. PLS-DA builds regression models to correlate the information in the X block (*i.e.*, raw data) to binary Y variables (*i.e.*, groups, class membership, *etc.*) by using the PLS algorithm (26). This approach was utilized to maximize the covariance between the independent variables X ‎ and the corresponding dependent variable Y (20). During model optimization, different preprocessing techniques‎ were applied. The optimal number of factors also known as latent variables‎ was selected based on the root mean square error of cross-validation (RMSECV) during cross-validation. In this case, RMSECV was plotted against the number of factors and the optimum number of factors that minimized the cross-validation error was selected.

*k-nearest neighbors algorithm*

*K*-NN as a pattern recognition technique was used for the classification of the samples. This algorithm attempts to categorize a new sample by computing the distance of that sample to all of the samples in the data matrix related to the training set (27). The predicted class of an unknown sample depends on the class of its *k* nearest neighbors. This model was applied following different data transforms and preprocessing methods mentioned before. During running the *k*-NN model, the Euclidean distance that separates each pair of samples in the training set was calculated in the pirouette software. Following running the process, the optimal k value with the lowest validation error was selected. 

*Model validation*

**Figure 1.**
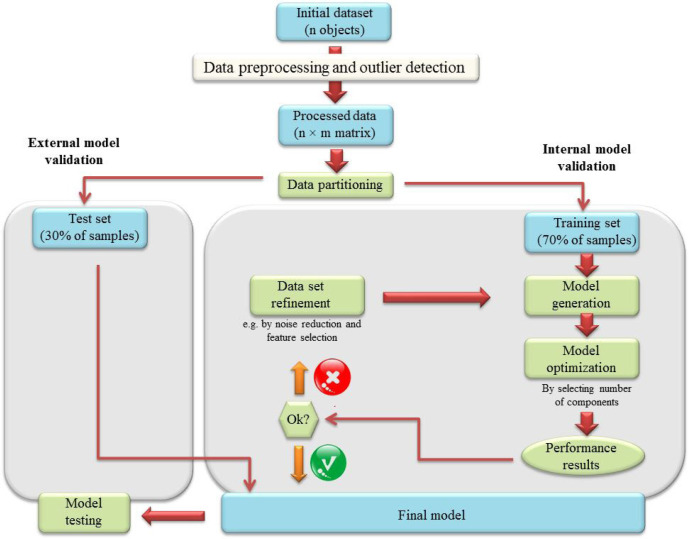
A detailed workflow of data analysis

To evaluate the performance of generated models, internal and external validations were performed on two different data sets. For this purpose, the initial dataset was divided into two subsets of 70% and 30% by performing Kennard-stone algorithm. Forty uniformly distributed samples (22 genuine and 18 adulterated juices) were placed in the training set and 16 samples (9 genuine and 7 adulterated juices) were in the test set. By performing the data partitioning, the knowledge of training dataset did not affect the test dataset and the predictive power of the created model increased subsequently (28). Leave-one-out cross-validation was applied on the training set for internal validation and the test set was used to externally validate the generated models. Data analysis was performed using Pirouette 4.5 software (Infometrix, Seattle, USA). A detailed workflow of data analysis is illustrated schematically in Figure 1. 

erated models several parameters including sensitivity, specificity, accuracy, and precision (Equations 1 to 4) were calculated. Matthew’s correlation ‎coefficient (MCC) and kappa value were also compared across PLS-DA and k-NN models using the following equations (Equations 5 and 6). In equations 1 to 6, TP, TN, FP, FN, P_0, _and P_e_ refer to true positive, true negative, false positive, false negative, the relative observed agreement among raters, and the hypothetical probability of chance agreement, respectively (29, 30).

Sensitivity = $\frac{\mathrm{TP}}{\mathrm{TP}+\mathrm{FN}}$

(Equation 1)

Specificity = $\frac{\mathrm{TN}}{\mathrm{TN}+\mathrm{FP}}$

 (Equation 2)

Accuracy = $\frac{\mathrm{TP}+\mathrm{TN}}{\mathrm{TP}+\mathrm{TN}+\mathrm{FP}+\mathrm{FN}}$

 (Equation 3)

Precision = $\frac{\mathrm{TP}}{\mathrm{TP}+\mathrm{FP}}$

 (Equation 4)

MCC = $\frac{\mathrm{TP}\times \mathrm{TN}-\mathrm{FP}\times \mathrm{FN}}{\sqrt[{2}]{\left(\mathrm{TP}+\mathrm{FP}\right)\left(\mathrm{TP}+\mathrm{FN}\right)\left(\mathrm{TN}+\mathrm{FP}\right)(\mathrm{TN}+\mathrm{FN})}}$

(Equation 5)

Kappa = $\frac{P_{0}-P_{e}}{1-P_{e}}$

 (Equation 6)

## Results

**Figure 2:**
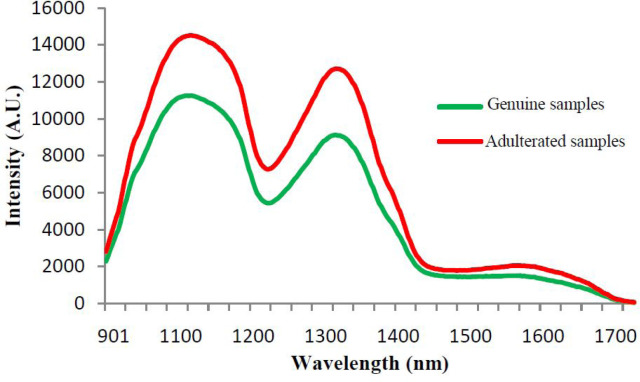
Mean NIR reflectance spectra of genuine and adulterated lime juice samples in the 900–1700 nm region

All genuine and adulterated samples were analyzed in triplicate and the average of three reflectance spectra was used for data elaboration. Mean NIR reflectance spectra of genuine and adulterated lime juice samples in the 900–1700 nm region are presented in Figure 2.

Confusion matrices of PLS-DA and *k*-NN models are presented in Tables 1 and 2. 

To evaluate the performance of the generated models, several performance parameters including sensitivity (true positive rate), specificity (true negative rate), accuracy, and precision were calculated. MCC and kappa value also were compared across PLS-DA and *k*-NN models. Values for each parameter in the internal validation and external validation sets are given in Table 3. 

## Discussion

**Figure 3:**
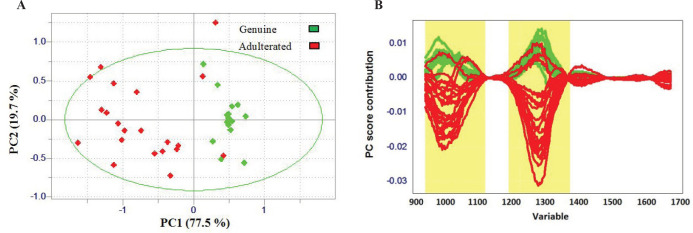
(A) PCA score plot of all genuine and adulterated samples with PC1 and PC2. (B) PC score plot of first PC; Wavelength regions with apparent separation power are highlighted

The present work is the first study that focuses on the capability of a handheld NIRS (Tellspec®) and chemometrics approach in the ‎detection of lime juice adulteration. This technology requires minimal equipment and user operation and offers ‎significant advantages over traditional platforms such as good speed and control, low cost, and ease-of-operation. Therefore, it can be used as a powerful lab-on-smartphone platform for the detection of lime juice adulteration. In this study, the correlation between measured multivariate spectral features (reflectance values of samples measured at different wavelengths) and the nature of samples (genuine or adulterated) was determined by performing unsupervised and supervised algorithms. PCA as a very popular technique for compression of data set was used to reduce the amount of data present in the pretreated spectra and to get a better overview of the data (32). As shown in Figure 3A, PC1 by 77.5 % and PC2 by 19.7% of the spectral variation explain most of the total variance in the samples. Although it seems that PCA is able to generate two separate clusters, some adulterated samples are still among the genuine ones. Loading plot of the first components (Figure 3B) demonstrates that the highest loading is around 901-1100 nm and 1200-1400 nm. Since the second overtone of O-H group is located in the 900–1000 nm region, water content affects this region (33, 34). The water content probably has a significant role in distinguishing adulterated lime juice samples from the genuine ones. 

**Figure 4:**
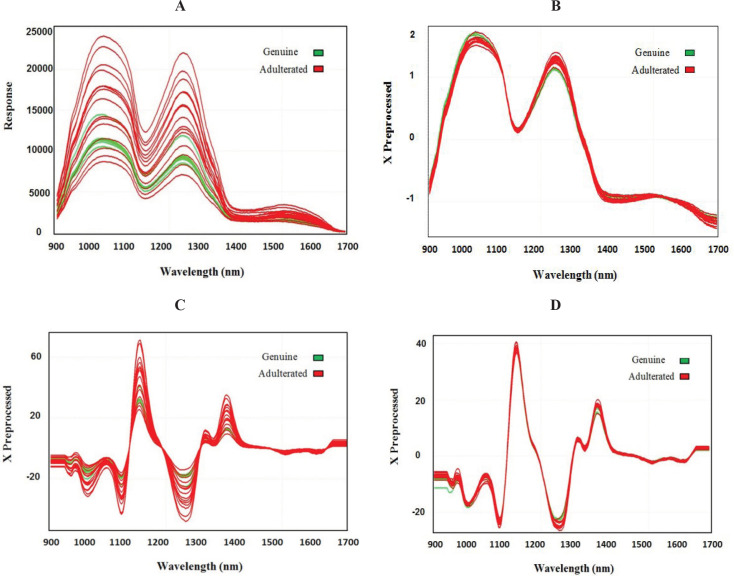
(A) Spectra of raw data and (B) different preprocessing techniques including SNV, ‎(C) second order derivative, and (D) MSC followed by second order derivative

PLS-DA was performed in order to sharpen the separation between genuine and adulterated samples. For this purpose, raw intensity values from the NIR sensor were subjected to different preprocessing prior to developing the PLS-DA model and the most accurate classification with 95% accuracy in internal validation and 88% accuracy in external validation was obtained with SNV preprocessing (Table 1). SNV pre-processing, which is one of the most applied methods of NIR data (35), helped remove the interferences of scattering, particle size, and the change of light distance (Figure 4). The complexity of a predictive model is defined by the number of factors. Since the importance of a factor in the prediction model is indicated by the amount of variance explained by that factor, selecting the optimal number of factors is one of the most important steps in modeling. Selecting too many factors will result in an over-fitted model. It should be noted that an over-fitted model which includes unneeded predictors will lead to worse predictions in the feature (36). In this model, two factors were used for each genuine or adulterated class.

The performance of *k*-NN revealed that this model is able to classify adulterated and genuine lime juice with an accuracy of 95% and 88% in the internal and external validation sets, respectively. This classification rate was achieved with MSC followed by 2^nd^-Dv preprocessing which are probably the most widely used techniques for NIR data. MSC and 2^nd^-Dv were used to remove artifacts or imperfections such as undesirable scatter effect and bring out the ‎spectra features (Figure 4). In the *k*-NN model, parameter k has an important influence on the classification model (14). Therefore, several k values were used to calculate the prediction potential of the model, and the best k value was found to be 5.

Assessing and analyzing the outputs of learning algorithms and finally interpreting this analysis are very critical steps in evaluating the performance of different learning algorithms especially when the sample size is partly small (29). To assess and interpret the result of generated classification algorithms in this study, they were evaluated in several ways. Sensitivity and specificity represent the correctly classified adulterated samples to the total number of adulterated samples and the correctly classified genuine samples to the total number of genuine samples, respectively. Although higher sensitivities in the internal validation and external validation were obtained with PLS-DA model (94% and 86%, respectively), *k*-NN delivered higher specificity in both internal validation (100%) and external validation (100%). This higher specificity in *k*-NN model could probably be related to the groups which were not the same size. *K*-NN classifier also favors the bigger group. Accuracy which refers to the percentage of total correct predictions is one of the most commonly used parameters for the evaluation of classification performance (37). There was no significant difference between the accuracy of PLS-DA and *k*-NN in the internal validation set (95% for both classifiers) and test set (88% for both classifiers). Another factor for evaluation the model performance is precision which shows the proportion of correctly classified adulterated samples to the total number of adulterated predicted samples (37). In this study, the *k*-NN model delivered the highest precision in both internal validation and test sets. 

MCC metric (ranging from -1 to +1) represents a correlation coefficient between the observed and predicted classifications. Since MCC takes into account all four classifying metrics (TP, TN, FP, and FN), it is a suitable metric for imbalanced data (38). The values equal to +1 represent a perfect prediction while -1 shows the worst possible prediction (29). PLS-DA with the MCC value of 0.90 and 0.75 in the internal and external validation, respectively, illustrated almost similar performance compared to that of the *k*-NN model (MCC value of 0.90 in the internal validation and 0.76 in the external validation). 

Kappa statistic, which ranges from +1 to -1, is a comprehensive single value based on the contingency table which takes into account the possibility of the agreement occurring by chance (38, 39). PLS-DA and *k*-NN models delivered a near value of kappa to +1, indicating a very good concordance of the models’ prediction and the actual classes. The results of this preliminary study showed that the method based on handheld NIR data is promising as ‎ a screening method for this type of adulteration. We should indicate that the difference found in the performance of the two models could be due to the limited number of samples in the current study. Therefore, analyzing more samples for a better estimation of the models’ performance and an increase in ‎the robustness of the generated models is highly recommended. 

**Table 1:** The confusion matrix of PLS-DA model

	**Target Class (Training)**					**Target Class (Test)**			
		**0**	**1**	**All**			**0**	**1**	**All**
**Output Class ** **(Training)**	0	17 42%	1 3%	94% P 6% F	**Output Class ** **(Test)**	0	6 38%	1 6%	86% P 14% F
	1	1 3%	21 52%	95% P 5% F		1	1 6%	8 50.0%	89% P 11% F
	All	94% P 6% F	95% P 5% F	95% P 5% F		All	86% P 14% F	89% P 11% F	88% P 12% F

0 = adulterated samples, 1 = genuine samples, P = pass, F = fail, Output class = PLS-DA output class, Target class = Real data class.

**Table 2:** The confusion matrix of *k*-NN model

	**Target Class (Training)**					**Target Class (Test)**			
		**0**	**1**	**All**			**0**	**1**	**All**
**Output Class ** **(Training)**	0	16 40%	0 0%	100% P 0% F	**Output Class ** **(Test)**	0	5 31%	0 0%	100% P 0% F
	1	2 5%	22 55%	92% P 8% F		1	2 13%	9 56%	82% P 18% F
	All	89% P 11% F	100% P 0% F	95% P 5% F		All	71% P 29% F	100% P 0% F	88% P 12% F

0 = adulterated samples, 1 = genuine samples, P = pass, F = fail, Output class = *k*-NN output class, Target class = Real data class.

**Table 3:** Classification results of internal and external validation

Settings	Internal validation		External validation	
	PLS-DA	*k*-NN	PLS-DA	*k*-NN
	2 Factors	K = 5	-	-
Pre-processing	SNV	MSC + 2^nd ^- Dv	-	-
Sensitivity (TPR)	0.94	0.89	0.86	0.71
Specificity (TNR)	0.95	1.00	0.89	1.00
Accuracy	0.95	0.95	0.88	0.88
Precision	0.94	1.00	0.86	1.00
Matthew’s CC	0.90	0.90	0.75	0.76
Kappa	0.90	0.90	0.75	0.74

## Conclusion

In this study, the feasibility of NIR spectroscopy and chemometrics approach in the discrimination of genuine and adulterated lime juices was investigated. A close relationship between NIR spectra and lime juice purity was found during data analysis. This study has revealed for the first time that NIRS (900-1700 nm) and machine learning methods such as PCA, PLS-DA, and *k*-NN could be applied for rapid detection of adulterated lime juices. PLS-DA and *k*-NN models were able to detect water and citric acid adulterated lime juices. Wavelengths around 901-1100 nm and 1200-1400 nm which were related to the O-H group of water had a significant role in distinguishing adulterated lime juice samples from the genuine ones. Generally, results of this study provided empirical evidence of the potential of the handheld near infra-red ‎spectrometer and machine learning methods for rapid detection of lime juice adulteration. Therefore, it ‎could be considered as an indicator of the total method performance for this application‎. Portable NIRS with an appropriate multivariate calibration model could also be used for the rapid detection of adulterated lime juices by industry and regulatory perspectives. However, since a limited number ‎of samples were used in the current study, more samples from a wider range of variability are required to increase the robustness of the ‎generated models and to confirm the promising results achieved in this study. It is highly recommended to generate the other models with selected variables to achieve more of the desired results.
